# Monozygotic Triplets and Dizygotic Twins following Transfer of Three Poor-Quality Cleavage Stage Embryos

**DOI:** 10.1155/2012/763057

**Published:** 2012-12-24

**Authors:** Reshef Tal, Dmitry Fridman, Richard V. Grazi

**Affiliations:** Division of Reproductive Endocrinology and Infertility, Maimonides Medical Center, No. 1355, 84th Street, Brooklyn, NY 11219, USA

## Abstract

*Background*. Assisted reproductive technology has been linked to the increased incidence of monozygotic twinning. It is of clinical importance due to the increased risk of complications in multiple pregnancies in general and in monozygotic twins in particular. *Case*. A 29-year-old female, nulligravida underwent her first IVF cycle. Three poor-quality cleavage stage embryos were transferred resulting in monochorionic triamniotic triplets and dichorionic diamniotic twins. Selective embryo reduction was performed at 12 weeks leaving dichorionic twins. The patient underwent emergency cesarean section due to preterm labor and nonreassuring fetal heart tracing at 30 weeks of gestation. *Conclusion*. Our case emphasizes that even embryos with significant morphological abnormalities should be considered viable and the possibility of simultaneous spontaneous embryo splitting must be factored into determining number of embryos to transfer.

## 1. Introduction

Although assisted reproductive technology (ART) has improved the chances of many subfertile couples to achieve pregnancy, it has increased the occurrence of multifetal pregnancies, most of which are dizygotic twins due to the transfer of more than one embryo. ART has also been linked to the increased incidence of monozygotic twinning, which is of clinical importance due to the increased risk of complications in multiple pregnancies in general, and in monozygotic twins in particular. Multifetal pregnancies are characterized by increased risk of both fetal and maternal complications. Fetal complications include restricted growth, pregnancy loss, preterm delivery, and perinatal mortality. Maternal complications include increased nausea and vomiting, gestational hypertension, and preeclampsia. In addition, monozygotic twin pregnancies carry specific risks, depending on their amnionicity, including twin to twin transfusion syndrome and cord entanglement. Numerous studies indicate that monozygotic twinning rates following ART procedures are between two and twelve times higher than the natural occurrence of 0.4% [1–4]. Several cases of monozygotic triplets as a result of ART procedures have also been reported [5–10]. The reason for the increased incidence of monozygotic twinning after ART has been a matter of debate for a long time and there is no definite explanation. However, various factors have been shown to be associated with monozygotic twinning. These include techniques in which there is zona pellucida manipulation such as intracytoplasmic sperm injection (ICSI) [11, 12] or assisted hatching [13, 14], and also after frozen embryo transfer [1] and/or blastocyst transfer [15, 16].

Here, we report a case of quintuplet pregnancy consisting of monozygotic (monochorionic triamniotic) triplets and dizygotic (dichorionic diamniotic) twins following transfer of three cleavage stage embryos. Remarkably, it occurred despite the poor quality of the transferred embryos and in the absence of any of the aforementioned risk factors for monozygotic twinning.

## 2. Case Report

**Figure 1:**
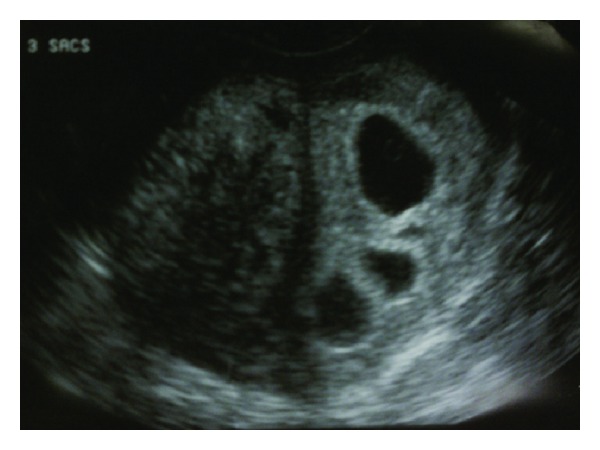
Three gestational sacs in the 5th week of gestation.

**Figure 2:**
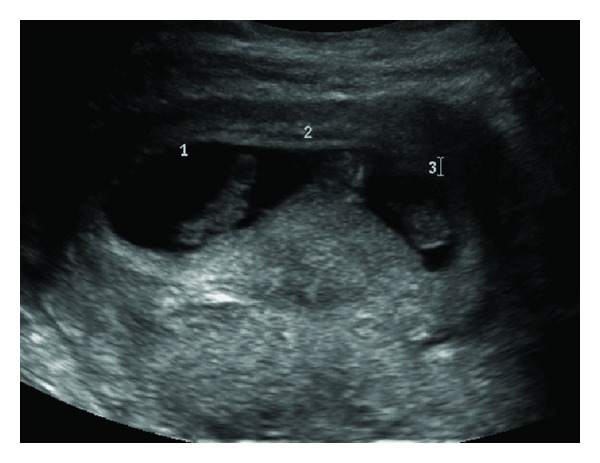
The monozygotic (monochorionic triamniotic) triplets in the 7th week.

An IVF cycle was planned for a 29-year-old nulligravida patient with unexplained infertility. Prior to this, she had one failed intrauterine insemination (IUI) and three failed IUI cycles with clomiphene citrate. The stimulation was performed with recombinant FSH and hMG using a short protocol with GnRH antagonist. Fourteen oocytes were aspirated during follicular puncture. Seven oocytes were fertilized and cultured. The cultured embryos were of relatively poor quality and the grading of the three embryos selected for transfer on day 3 was 6-cell, grade C; 4-cell, grade B; 4-cell grade C [17]. About 4 weeks later, three intrauterine gestational sacs were noted by transvaginal ultrasound (Figure 1). At 7 weeks of gestation, a quintuplet pregnancy with monozygotic (monochorionic, triamniotic) triplets and dizygotic (dichorioinc diamniotic) twins was sonographically confirmed (Figure 2). All five embryos demonstrated cardiac activity and were concordant. The patient was counseled and referred for selective fetal reduction of the monochorionic triamniotic triplets. This was performed without complications on the 12th week of her pregnancy. Amniocentesis of the triplets during fetal reduction showed normal chromosomes (46, XX) and no structural abnormalities. The pregnancy was subsequently complicated by preterm labor followed by emergency cesarean delivery due to nonreassuring fetal tracing resulting in delivery of healthy female and male babies at 30 weeks of gestation. Both babies were born in 2006 and are currently doing well.

## 3. Discussion

A major current focus in the field of ART has been on the reduction of multifetal gestation rates. One way of achieving this is the selection of one or a maximum of two embryos after prolonged culture for 5-6 days. It has been shown that, in “good prognosis” patients, the rate of dizygotic twinning can be substantially reduced while maintaining robust pregnancy rates by adapting a single embryo transfer (SET) policy [18]. However, accumulating data suggest increased risk of monozygotic twinning following IVF, especially after prolonged culture and blastocyst transfer [1, 19]. This lies in contrast with the aim of extended culture: the selection of a single blastocyst to achieve a single pregnancy.

Monozygotic twinning is an uncommon phenomenon, the etiology of which is still unclear. Monozygotic twinning rates following ART procedures are between two and twelve times higher than the natural occurrence of 0.4% [1–4]. Monozygotic twins carry additional risks above those of multiple pregnancies including higher perinatal morbidity and mortality, and increased risk of developmental anomalies, discordant growth, and prematurity. The incidence of monozygotic triplets is much more rare (~0.000023%) [20].

In addition to prolonged culture and blastocyst transfer, monozygotic twinning has been associated with several other risk factors. Disruption of the zona pellucida that occurs during ICSI was shown to lead to increased rate of monozygotic multiples in several studies [11, 12, 19]. Another form of zona pellucida manipulation which could lead to an increased rate of monozygotic multiples is assisted hatching [13].

While there is some disagreement as to the impact of maternal age on monozygotic twinning rate, a 12%–22% increase in MZ twinning has been reported in women over the age of 35 compared with women younger than 25 [21]. More recent reports indicate a trend of increasing monozygotic twinning following IVF associated with advanced maternal age [19, 22]. On the other hand, other studies found no association between maternal age and monochorionic twinning [12, 23]. These limited data indicate that increasing maternal age may contribute to an increased incidence of MZ twins, but the contribution is likely minimal in ART cases.

Another factor that has been linked to monozygotic twinning is transfer of frozen embryos [8, 9]. A recent meta-analysis study showed a monozygotic twinning rate of 3% following frozen embryo transfer but the small number of cases precluded the authors from reaching statistical significance [1].

In this case paper, we describe the occurrence of a quintuplet gestation consisting of monozygotic (monochorionic triamniotic) triplets and dizygotic (dichorionic diamniotic) twins following transfer of three poor-quality cleavage stage embryos. Previously, several cases of monozygotic triplets following IVF have been reported in which ICSI [5, 7, 10], assisted hatching [5, 7], blastocyst transfer [6, 9], or frozen embryo transfer [8, 9] were the risk factors for the monozygotic triplet occurrence. It is noteworthy that in contrast to these case reports, in our case monozygotic splitting into triplets occurred without any of the known risk factors previously discussed. One similar case report to ours was found in the literature. Salat-Baroux et al. described a case of trizygotic quintuplets (monoamniotic triplets with two additional fetal sacs) following IVF and the transfer of four grade 1 embryos without zona pellucida manipulation or extended culture [24]. Our case differs from their case report in that we transferred a smaller number of embryos and they were all of poor quality.

The etiology of the quintuplet gestation described here is difficult to determine. Edwards et al. [2] suggested that the nature of embryonic growth *in vitro* predisposes to twinning. A possible “hardening” of the human zona *in vitro* after exposure to artificial media, as opposed to salpingeal or uterine secretions, could lead to increased fragility or brittleness of the zona pellucida, or cell-to-cell adhesion might be disturbed after *in vitro* culture. In agreement with this, Cassuto et al. showed that improved culture media may reduce the incidence of monozygotic twins [25]. Alternatively, Schachter et al. [26] suggested that improved endometrial conditions after gonadotropin therapy may encourage monozygotic implantation, or that the biochemical milieu of the uterine cavity after gonadotropin therapy encourages asymmetrical ZP hatching, independent of zona manipulation procedures done *in vitro*.

In conclusion, the current paper emphasizes that monozygotic triplets are possible consequence of ART even in cycles where the known risk factors for monozygotic twinning do not exist, and it is, therefore, necessary to identify additional predictive factors for their occurrence. This can be accomplished by performing large case-control studies that will allow us to counsel patients appropriately. In addition, the present case demonstrates that even cleavage stage embryos with significant morphologic abnormalities should be considered viable with implantation potential, underlining the importance of minimizing the number of embryos transferred and moving towards single embryo transfer in IVF.
